# Reliability of Ultrasound Features in Predicting Thyroid Malignancy

**DOI:** 10.7759/cureus.94870

**Published:** 2025-10-18

**Authors:** Sushmitha D J, Rahul A V, Kiran Shankar

**Affiliations:** 1 Surgical Oncology, Cauvery Heart and Multi-Speciality Hospital, Mysuru, IND; 2 Medicine, Maurya Multispeciality Hospital Mysuru, Mysuru, IND

**Keywords:** onco-surgery, radio diagnosis, thyroid cancer surgery, thyroid nodule size, ultrasound neck

## Abstract

Introduction:* *Thyroid nodules have shown an increasing trend over the past years, with a recent surge. Ultrasound is the primary modality of investigation to detect malignancy in thyroid nodules, which is safe, cost-effective, and easily accessible, whereas* *fine needle aspiration biopsy is an invasive and costly test with risk factors. This study aims to assess how accurately a predefined five-feature ultrasound (US) composite (microcalcifications, marked hypo echogenicity, irregular/micro lobulated margins, intranodular vascularity, and suspicious lymph nodes) can identify disease compared to histopathology by measuring its sensitivity, specificity, positive predictive value (PPV), negative predictive value (NPV), and area under the curve (AUC) with 95% confidence intervals. The ultrasound composite is defined a priori as positive if two or more of the five features are present. The secondary objective is to explore which smaller combination of features provides the best diagnostic performance based on ROC analysis.

Methodology: Between August 2015 and December 2022, 313 consecutive patients with thyroid nodules presenting to the Surgical Department were screened; out of these, 146 patients underwent surgery* *for the presence of large size, suspicious swelling, and other cosmetic reasons. All patients underwent routine preoperative fine needle aspiration cytology (FNAC) and neck ultrasound. Final histopathology comprised the diagnostic accuracy analysis set (per-nodule analysis of the surgically excised nodule). Ultrasound features and FNAC results were correlated with the final histopathological report. US features recorded were hypoechogenicity, irregular margin, microcalcification, increased vascularity, and a suspicious lymph node. Data was statistically analyzed using the multiple logistic regression method, the receiver operating characteristic (ROC) curve, the cross-tabulation McNemar’s chi-square test, and IBM Corp. Released 2012. IBM SPSS Statistics for Windows, Version 18. Armonk, NY: IBM Corp. We prespecified the diagnostic rule a priori as US-positive if ≥2 of the five features were present. Diagnostic performance measures were calculated against histopathology.

Results: Using ultrasound diagnostic criteria, 112 patients were classified as positive, and histopathology later confirmed that 84 of these had malignancies. This showed a sensitivity of 95.45%, a specificity of 51%, and a positive predictive value of 75%. Further analysis via the ROC curve showed a high malignancy probability at a point that corresponds to the presence of the 2 ultrasound characteristics, i.e., irregular margin and microcalcification. Specificity and sensitivity at this point are 73.5 and 74.4, respectively.

Conclusion: The predefined rule using ≥2 features showed high sensitivity for detecting malignant thyroid nodules, making it useful for screening. The presence of irregular margins and microcalcifications (M + C pair) provided a more balanced sensitivity-specificity trade-off, supporting its use for risk stratification rather than direct surgical decision-making. While these ultrasound criteria show strong diagnostic performance, definitive management decisions should continue to rely on multidisciplinary assessment, integrating cytology, clinical findings, and patient factors. Further prospective validation in larger, unselected cohorts is recommended to refine threshold criteria and confirm generalizability.

## Introduction

Thyroid nodules are highly prevalent in clinical practice. The American Thyroid Association defines a thyroid nodule as a discrete lesion radiologically distinct from the surrounding parenchyma. The prevalence of thyroid nodularity varies from 19% to 67% and increases with age, affecting 50% of the population older than 40 years [1]. Malignant nodules are detected in only 5-15% of cases [1]. The incidence of thyroid cancer has increased 2.4 times over the last 30 years. Many studies have validated the recent increase in the incidence of thyroid nodules [1-28] and the extreme need to exclude malignancy. Recent work has shown a shift from describing ultrasound features individually to using structured TI-RADS systems, which makes reporting more consistent [13]. Studies comparing ATA, BTA, and TI-RADS suggest they all have good sensitivity and negative predictive value, although their biopsy cut-offs are not the same [16]. Other reports have also shown that TI-RADS on its own can reliably predict the risk of cancer in everyday practice [23].

Thyroid ultrasound is the most sensitive method for the detection of nodular thyroid disease [6]. USG of the thyroid is safe, cost-effective, and easily accessible. Sonographic features of potentially malignant thyroid nodules include microcalcification, marked hypogenicity, irregular or microlobulated margins, and intranodular vascularity [9]. If the nodule has highly suspicious ultrasound features or clinical risk factors, surgical excision should be considered for definitive diagnosis [12].

Fine-needle aspiration biopsy, the gold standard for detecting malignancy, is an invasive and costly test with risk factors [5]. Thyroid FNA biopsy accurately classifies most nodules, but a significant proportion (20-30%) lacks cytological features for definitive classification [28]. From 2007 to now, the Bethesda Classification System for reporting thyroid FNAC has been used worldwide [29]. These include indeterminate cytology (AUS/FLUS) and (FN/HCN). From this perspective, the US not only determines the need for FNAC but can also play a crucial role independently in assessing the risk of malignancy in thyroid nodules, and recent studies have confirmed and validated the role of US in FNAC inconclusive results [28-32]. From this perspective, the US not only determines the need for FNAC but can also play a crucial role independently in assessing the risk of malignancy in thyroid nodules if the diameter of the nodule is large and there is calcification [15].

In the past, patients with thyroid cancer were invariably treated with partial or total thyroidectomy. Previously, the usual practice was to perform a diagnostic lobectomy in patients with these types of nodules. However, approximately 80% were ultimately found to be benign; hence, surgical lobectomy is no longer considered ideal for all cytologically indeterminate nodules [12]. In contemporary surgical practice, an increasing number of patients are treated with partial or total thyroidectomy [2]. Therefore, accurate guidance in decision-making is very important for preventing unnecessary procedures and second surgeries [2]. Recently, several studies have sought to determine the diagnostic validity of ultrasound in thyroid cancer by reporting a few USG features that were highly suggestive of malignancy, with a mean sensitivity of > 70% in all [1-9]. Many studies have compared various risk-stratification systems [10-25]. Although they lack information regarding US features correlating with specific types of cancer, it is inconclusive to plan the primary treatment modality.

This retrospective test aims to test the diagnostic accuracy of the preoperative ultrasound by correlating it with the final histopathology report.

## Materials and methods

This retrospective study was conducted at the Department of Surgical Oncology and Radiology of a tertiary care hospital. The data was collected from the hospital's electronic system, PACS (Picture Archiving and Communication System). The Institutional Review Board approval was obtained for this study.

Between August 2015 and December 2022, 313 patients with thyroid swelling were presented to the surgical department. All patients underwent a routine ultrasound of the neck and FNAC before thyroidectomy. The patients comprised 244 females and 69 males with a mean age of 42.09. Clinical assessment of all patients included history-taking and general and local examinations. A total of 146 patients were surgically treated for suspected ultrasound, large size, suspicion of malignancy, and other cosmetic reasons, and ultrasound was performed with an ultrasound scanner equipped with a 5-12 MHz linear array transducer. Adult patients (≥18 years) with at least one thyroid nodule and a complete ultrasound report documenting the five predefined suspicious features, irregular margin (M), microcalcification (C), hypo echogenicity (H), increased vascularity (V), and suspicious cervical lymph node (L), and with definitive histopathology following surgery were included (n-Nodule). Primary analysis was conducted on a per-nodule basis for the surgically excised/index nodule. For patients with multiple nodules, the index lesion was defined as the nodule that underwent excision (or, if more than one nodule was removed, the dominant/suspicious nodule as reported by the surgeon/pathology). The grayscale method was used to assess benign and malignant features. Scanning was performed in both transverse and longitudinal planes. Color Doppler technology was used to assess vascularity. Vascularity was considered positive only when intranodular or mixed (types 3-4) flow was observed on Doppler ultrasound, whereas absent or purely perinodular flow (types 0-2) was classified as negative.

Ultrasound (US), fine-needle aspiration cytology (FNAC), and final histopathology findings were included in the diagnostic accuracy analysis set. The analysis was performed per nodule, based on surgically excised nodules. Each nodule was considered an independent unit for assessment to ensure accuracy in evaluating diagnostic performance. Table 1 shows the definitions. 

**Table 1:** Definitions table

Term	Definition
Thyroid Nodule	A discrete lesion radiologically distinct from the surrounding thyroid parenchyma.
Prevalence of Thyroid Nodularity	The frequency of thyroid nodules in the population, varying from 19% to 67% and increasing with age.
Malignant Nodules	Thyroid nodules that are cancerous, detected in only 5-15% of cases.
Thyroid Ultrasound	A safe, cost-effective imaging method used to detect nodular thyroid disease with high sensitivity.
Sonographic Features	Characteristics observed on ultrasound that can indicate malignancy, including microcalcification, hypoechogenicity, irregular/microlobulated margins, and intranodular vascularity.
Fine Needle Aspiration Biopsy (FNAB)	A diagnostic procedure using a thin needle to extract cells for examination, considered the gold standard for detecting thyroid malignancy.
Bethesda Classification	A system for reporting thyroid FNAB results, including categories such as AUS/FLUS (Atypia/FLUS) and FN/HCN (Follicular Neoplasm/Hurthle Cell Neoplasm).
Total Thyroidectomy	Surgical removal of the entire thyroid gland.
Diagnostic Lobectomy	Surgical removal of part of the thyroid gland for diagnostic purposes, often replaced by partial or hemi-thyroidectomy.
Partial/Hemi-Thyroidectomy	Surgical removal of part of the thyroid gland, preferred in contemporary practice to avoid unnecessary procedures.
Sensitivity	The ability of a test to correctly identify those with the disease.
Specificity	The ability of a test to correctly identify those without the disease.
Positive Predictive Value (PPV)	The likelihood that a positive test result correctly indicates the presence of disease.
Negative Predictive Value (NPV)	The likelihood that a negative test result correctly indicates the absence of disease.

The ultrasound parameters assessed in all nodules were 1) hypogenecity, 2) irregular margin, 3) vascularity, 4) microcalcification, and 5) lymph nodes (Table 2). Thyroid nodules were classified as positive or negative based on ultrasound diagnostic criteria. The presence of two or more of the above-mentioned ultrasound features formed the basis of the criteria. That is “+” if any one of the above-mentioned two or more features was present. Sensitivity, specificity, positive predictive value, negative predictive value, and accuracy were calculated for individual sonographic characteristics, correlating them with the final histopathological results.

**Table 2:** Defining each ultrasound feature (we selected the above-mentioned five ultrasound characteristics based on their proven associations with the malignancy risk of thyroid nodules, as highlighted in the study). These features are commonly observed in malignant thyroid nodules and have been consistently linked to the accurate differentiation between benign and malignant conditions) [21]

Ultrasound Features	Definition
Hypogenecity (H)	The echogenicity of thyroid nodule refers to its brightness relative to normal thyroid parenchyma. The solid component is considered in predominantly solid/cystic nodules to check for echogenicity. Mildly hypoechoic refers to an appearance darker than the normal surrounding thyroid parenchyma but less dark than the surrounding strap muscle [18]. Nodule is termed as “markedly hypoechoic” if it is of lower echogenicity than overlapping strap muscle, and is thus highly suspicious for malignancy [7].
Margin (M)	It describes the outline of the thyroid nodule [18]. Irregular is defined as obviously discernible but non-smooth edges with speculation /microlobulation [25]. It may be, spiculated; presence of 1/ more sharp angled spiculations microlobulated; the presence of 1/more smooth, focal round protrusion on the margin [18].
Microcalcification (C)	True micro calcifications correspond to Psammoma bodies and are multiple round echogenic foci around 1mm in size without posterior shadowing located in the solid component of the nodule. They are highly suggestive of malignancy [18].
Vascularity (V)	Color/Power Doppler US can be used to evaluate nodule vascularity [25]. It is classified into three types Type 1- absence of intranodular /perinodular flow Type 2-presence of perinodular /slight intranodular Type 3-presence of marked intranodular/slightly perinodular In this study, type 1 and 2 are considered as positive [18].
Lymph Node (L)	A US survey of the cervical lymph nodes should be performed on all patients with thyroid nodules, especially those with intermediate and high-risk ones [18]. Lymph nodes with any of the following features are regarded as suspicious cystic changes, echogenic foci (calcification), cortical hypergenecity (focal/diffuse), abnormal vascularity (peripheral/diffuse) [25]

During fine needle aspiration biopsy, patients were made to lie down in a supine position, with a pillow placed under the shoulder, neck extended, and adequately exposed. A 23-gauge needle and a 10 ml syringe were used to perform fine needle aspiration cytology (FNAC). Ultrasound guidance for FNAC was utilized only when thyroid swelling was small/inconclusive on initial FNAC. In partially cystic nodules, cystic fluid was aspirated, and FNAC was performed on the solid components only. FNAC results obtained from the pathology department were classified according to the Bethesda Classification. (I) Non-diagnostic, (II) benign/follicular lesion of undermined significance, (III) atypia/FLUS, (IV) follicular nodule suspicious of follicular neoplasm, (V) suspicious of malignancy, (VI) malignant. Histopathological examination post thyroidectomy: the specimen was sent to the pathology department for histopathological examination performed by one pathologist. Pathologically, they are classified as follicular and non-follicular neoplasms and their subtypes. This final histopathological report correlated with the clinical, radiological, and FNAC results obtained preoperatively.

Statistics

Data were analyzed using IBM Corp. Released 2012. IBM SPSS Statistics for Windows, Version 18. Armonk, NY: IBM Corp., M.C. chi-square test, cross-tabulations were used. The numbers of true positives (TP), true negatives (TN), false positives (FP), and false negatives (FN) were calculated. Sensitivity and specificity, positive predictive value (PPV), and negative predictive value (NPV) were calculated, and p-values less than 0.001 were considered statistically significant.

## Results

While conducting this study, 313 thyroid nodules were studied with a female-to-male ratio of around 1:0.2, and the mean age of the subjects was around 42.07.

**Figure 1:**
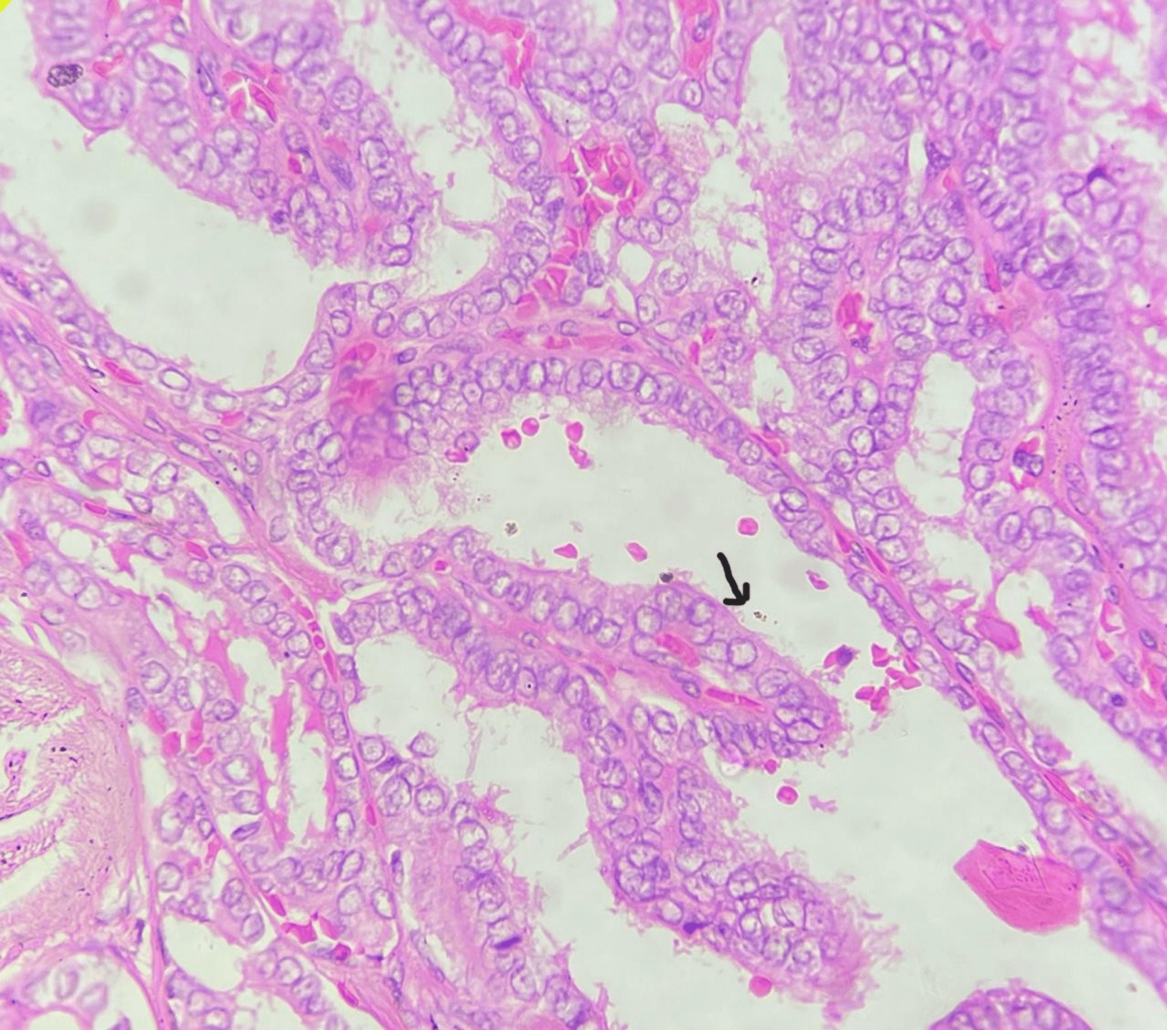
Histopathological confirmation: the biopsy reveals a predominantly papillary architecture with characteristic features of PTC, showing papillae covered with atypical cells exhibiting nuclear atypia with the ‘Orphan-Annie nuclei’ appearance.

Papillary carcinoma of the thyroid was the most common malignancy to be diagnosed on initial FNAC. Figure 1 shows the histopathological biopsy result of a 1.5 cm hypoechoic thyroid nodule with microcalcifications, which reveals a predominantly papillary architecture with characteristic features of PTC, showing papillae covered with atypical cells exhibiting nuclear atypia with the ‘Orphan-Annie nuclei’ appearance. However, 63.6% (200) and 25% (79) of the total thyroid nodules were papillary thyroid carcinoma and its follicular variant, respectively. Another type of cancer included 10.2% (31) of follicular thyroid carcinoma and 1.1% (3) of anaplastic thyroid carcinoma.

Preoperative U.S. neck in papillary thyroid carcinoma showed predominant features like increased vascularity (intra-nodular/peripheral) with sensitivity and specificity of 75% and 60%, respectively; calcification with sensitivity and specificity of 75% and 82%, respectively; and lymph nodal involvement on USG with the positive predictive value of 86.2%.

The histopathology result was correlated with the initial FNAC performed preoperatively. Bethesda Class 3, 4, and 5 constituted about 38% (119) of the entire population (Table 3).

**Table 3:** Diagnostic validity of preoperative FNAC (Bethesda 1-5) correlated with the final histopathology report. Data has been represented as number of nodules (N), number of malignant and benign lesions in the final histopathological report as (N), and percentage (%) of malignancy. A p-value is considered significant at <0.001 N: number of nodules, %: percentage

Bethesda Classification	Number of thyroid nodules (N)	Final Pathology		Percentage Malignancy (%)	p-value
		Malignant	Benign		
Benign /follicular lesion of undermined significance	8	3 (37.5%)	5 (62.5%)	37.5%	p <0.001
Atypia/FLUS	23	6 (26%)	17 (74%)	26%	p <0.001
Follicular nodule suspicious of follicular neoplasm	69	42 (60.86%)	27 (39.13%)	60.8%	p <0.001
Suspicious of malignancy	27	19 (70.37%)	8 (29.6%)	70.37%	p <0.001
Malignant	19	18 (94.7%)	1 (5.26%)	94.7%	p <0.001

Using the prespecified ≥2-feature rule, including the following features: irregular margin (M), microcalcification (C), vascularity (V), hypogenecity (H), and lymph node metastasis (L), 112 thyroid nodules were classified as positive. On final histopathology, 84 of 112 ultrasound-positive nodules were malignant and 28 were benign; among the 34 ultrasound-negative nodules, four were malignant and 30 were benign. This showed a sensitivity of 95.45%, a specificity of 51.72%, a positive predictive value of 75%, and a negative predictive value of 88.24% (p-value <0.001) (Table 4).

**Table 4:** Diagnostic accuracy of the prespecified ≥2-feature ultrasound rule for predicting thyroid malignancy (n = 146), "n" is the number of thyroid nodules

Diagnostic Metric	Formula	Value (%)	95% Confidence Interval (Exact/Wilson)
Sensitivity	84/(84 + 4)	95.45	88.9 – 98.2
Specificity	30/(30 + 28)	51.72	39.2 – 64.1
Positive Predictive Value (PPV)	84/(84 + 28)	75.00	66.2 – 82.1
Negative Predictive Value (NPV)	30/(30 + 4)	88.24	73.4 – 95.3
Accuracy	(84 + 30)/146	78.08	70.6 – 84.0
χ² test (Ultrasound vs Histology)	—	—	p < 0.001

The risk of malignancy increased with an increase in the US characteristics of S/o malignancy. The presence of all the suspicious US features was highly suggestive of malignancy (p-value <0.001).

The positive predictive value of microcalcification/intranodular vascularity and hypogenecity in the US was 79%, 86%, and 87%, respectively (p-value < 0.001).

Predictive of two ultrasound characteristics, i.e., irregular margin (M) and microcalcification (C), it shows the sensitivity of 74.4% and specificity of 73.5% (p-value <0.001).

**Figure 2:**
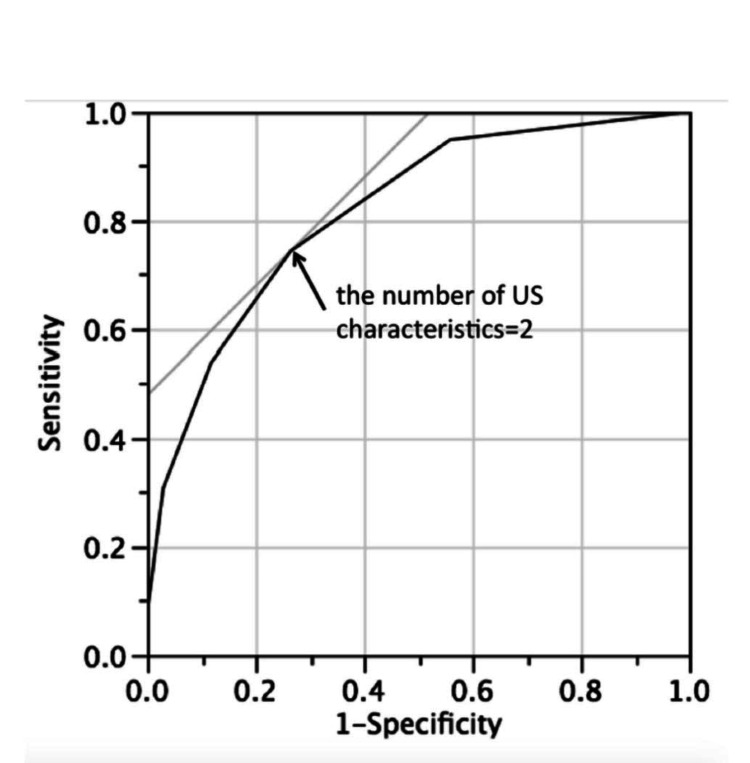
ROC curve showing the predictive value of thyroid malignancy based on the U.S. characteristics. In the above figure, the arrow shows the point where the number of ultrasound characteristics present is two. Data of sensitivity and specificity are represented as percentages (%). Statistical significance was considered at p<0.001. US: Ultrasound

The ROC curve showed a high probability of malignancy at the point marked with an arrow in Figure 2.

We conducted a prespecified secondary analysis evaluating the diagnostic performance of the M + C pair (irregular margin and microcalcification), as reported in Table 5. This pair demonstrated a sensitivity of 74.4% and a specificity of 73.5%, corresponding to the ROC threshold described earlier. Compared with the ≥2-feature diagnostic rule, which achieved higher sensitivity but lower specificity; however, the M + C pair offered a more balanced sensitivity-specificity trade-off. Accordingly, we propose using the ≥2-feature rule as the primary screening criterion to maximize malignancy detection, while the M + C pair may serve as a secondary confirmatory marker to enhance post-test probability and reduce false positives.

**Table 5:** Sensitivity and specificity of ultrasound characters in predicting malignancy. Data are presented as percentages. Statistical significance was considered at p<0.001. Ultrasound Characteristics—Irregular Margin (M), Hypogenicity (H), Microcalcification (C), Vascularity (V), and Lymph Node Metastasis (L), CI: Confidence interval; the 100% sensitivity and specificity observed for nodules with all five features reflect a very small sample size (n = 6) and should be interpreted with caution as a small-cell artifact rather than absolute diagnostic performance.

No. of ultrasound characteristics present (N)	Ultrasound features present	No. of nodules (n)	Malignant (n)	Benign (n)	Sensitivity % (95% CI)	Specificity % (95% CI)	p-value (Chi-square)
5	All (M, H, V, C, L)	6	6	0	100 (54.1–100)	100 (93.8–100)	<0.001
4	H, V, C, M	18	17	1	94.4 (72.7–99.9)	97.1 (88.8–99.9)	<0.001
3	V, C, M	34	29	5	85.3 (71.6–93.1)	88.2 (76.6–95.0)	<0.001
2	M, C	54	32	22	59.3 (47.2–70.4)	73.5 (60.4–83.9)	<0.001
1	M	22	4	18	18.2 (7.3–36.4)	44.1 (31.0–58.0)	<0.001
0	None	12	0	12	0 (0–25.9)	100 (93.8–100)	<0.001
Total	—	146	88	58	—	—	—

## Discussion

Thyroid ultrasonography is a common condition encountered in daily practice. The management of thyroid nodules is always a multidisciplinary approach, involving a team of radiologists, pathologists, and surgeons. Ultrasound is the initial step towards management, and a radiologist plays a crucial role in determining malignancy at a very early stage, influencing the choice of treatment modality.

Studies performed to date have shown that US characteristics when analyzed alone are very poor indicators of malignancy; however, when used in combination, they show higher malignancy ratios and probabilities [1-10]. Recent work has shown a shift from describing ultrasound features individually to using structured TI-RADS systems, which makes reporting more consistent [13]. Studies comparing ATA, BTA, and TI-RADS suggest they all have good sensitivity and negative predictive value, although their biopsy cut-offs are not the same [16]. Other reports have also shown that TI-RADS on its own can reliably predict the risk of cancer in everyday practice [23].” This study aimed to gain a better understanding of the reliability of US features in predicting malignancy by correlating them with the final histopathological diagnosis. Our study confirms that the US diagnostic criteria are accurate in predicting malignancy in thyroid nodules. The present results are consistent with those of Kim et al., who dealt with the effectiveness of US diagnostic criteria in predicting malignancy. Some recent studies cast light on scoring systems that can help radiologists label nodules to choose patients for FNAC [11-27].

Few studies have suggested using multiple sonographic features to categorize nodules as benign or malignant [1-10]. Nodule diameter and heterogeneous echotexture are often considered when assessing thyroid nodules, but the evidence in the literature is mixed. Some studies suggest that larger nodules or those with heterogeneous textures are more likely to be malignant, while others have shown that these features on their own are not specific enough to be reliable [2,3,9]. More recent work highlights that the real value lies in ultrasound features such as irregular margins and microcalcifications, which are stronger indicators of malignancy than size or heterogeneity [10,12,23]. For this reason, diameter and heterogeneity should be interpreted alongside other high-risk ultrasound features rather than being used in isolation.

In our study, US characteristics such as hypogenicity, irregular margins, increased vascularity, microcalcification, and lymph nodes were studied. Our results confirmed that the risk of malignancy increases with an increase in these US characteristics. These findings are in line with a study conducted in Japan, which used a multiple logistic regression approach similar to our study and proved that the five US characteristics are solid and hypoechogenic. Irregular shape, ill-defined margins, and microcalcifications were the most reliable predictors of malignancy in non-follicular neoplasms. Another promising finding was that the presence of two US characteristics, irregular margins and microcalcification, increased the risk of malignancy by 76%. In our study, irregular margins and microcalcifications emerged as the most reliable indicators of thyroid malignancy, a finding consistent with earlier reports and meta-analyses [5,11,20]. Remonti et al. and Brito et al. both demonstrated that these features have the highest specificity among sonographic predictors, while Horvath et al. incorporated them as key components of the thyroid imaging reporting and data system (TIRADS) risk stratification system. A similar pattern of results was obtained in 2011 by Frederico et al., suggesting that irregular margins and microcalcifications are the strongest predictors of malignancy in thyroid nodules [10]. Differences between studies may be due to variations in sample size, patient populations, and the subjective interpretation of ultrasound features. In comparison with the above studies, our diagnostic criterion supports existing evidence that the combination of suspicious features strengthens accuracy. In particular, irregular margins and microcalcifications consistently stand out as the most dependable predictors. This approach offers a practical and reliable method for identifying malignant thyroid nodules.

According to this, 112 (76.71%) out of 146 were classified as positive, of which 88 (60.2%) turned out to be malignant on final definitive diagnosis from histopathological reports. The present study proved that the quoted criterion is 95.45% sensitive and 51% specific, with a positive predictive value of 75%. The majority of thyroid nodules in our study included papillary thyroid carcinomas, accounting for 63.6% (200) of the total cases. Microcalcification, lymph node enlargement, and increased vascularity were the predominant features of the papillary thyroid carcinoma subtype in our study. This result highlights the little-known facts regarding the ultrasound-based diagnosis of papillary thyroid carcinoma. Overall, these findings are in accordance with the findings reported by Wong et al. [2], who reported that on FNAC, Bethesda 3, 4, and 5 categories constituted the most challenging group of nodules to categorize as malignant and perform surgery.

Recent literature suggests that the US plays a vital role in predicting malignancy in such nodules and determining the extent of surgical treatment [29-31]. Our study population comprised approximately 38% (119) of the nodules with indeterminate cytology. We recommend caution in translating these results to surgical practice. In patients with indeterminate cytology (Bethesda 3-5), a highly suspicious US pattern (≥2 features) may support a multidisciplinary discussion and could tip the balance toward more extensive surgery in selected cases, as the second surgery involves completion thyroidectomy and is associated with higher rates of transient/permanent hypocalcemia/recurrent laryngeal nerve injury. However, routine recommendation of total thyroidectomy solely based on retrospective US criteria is not supported without prospective validation, decision-curve analysis, and guideline comparison (e.g., TI-RADS/ATA). Furthermore, incorporating external beam radiotherapy and radioactive iodine therapy into the treatment plan for selected patients is essential for optimizing outcomes. External beam radiotherapy can serve as an effective adjunct to surgery and radioactive iodine (RAI) therapy, particularly in cases where gross residual disease remains after surgical intervention [33,34].

The strength of this study lies in robust correlative analysis. This analysis links the ultrasound features with the final histopathological outcomes, enhancing the predictive value of the diagnostic criterion. The high sensitivity of the US criterion that is being reported validated the effectiveness of the diagnostic criterion in detecting malignancy and also its practical implications for surgical decision-making. As the study is consistent with the existing literature, it strengthens the reliability of US characteristics. Although the present results support the idea of using US diagnostic criteria as one of the components of surgical decision-making, it is appropriate to recognize a few limitations of this study. Most thyroid nodules included in this study were papillary thyroid carcinomas, showing selection bias. Ultrasound readings can vary between observers and even for the same observer at different times. This shows the subjective nature of ultrasound interpretation while recognizing the ultrasound characteristics as quoted in the diagnostic criterion. A limitation of this study is that inter-reader reliability was not assessed, as only single-reader interpretations were available. Future prospective work should include blinded dual readings to quantify reproducibility using Cohen’s K. Using standardized systems like TIRADS and assessing observer agreement can help improve reliability in future studies. The personal interpretation of ultrasound features can result in different diagnoses by various radiologists. The study does not adequately address how to determine the appropriate extent of surgery beyond the presence of suspicious features. Despite such limitations, current research contributes to a growing body of evidence regarding the role of ultrasound in planning surgical treatment.

Incorporating a machine learning model could enhance the predictive power of the existing criteria, especially in terms of handling larger and more complex datasets. The use of machine learning algorithms such as random forests, support vector machines (SVM), or neural networks could be explored to better handle the nonlinear relationships between ultrasound features, clinical parameters, and histopathology, thereby increasing the robustness of the malignancy prediction model and providing the scope for future research.

## Conclusions

Finally, our study evaluated a predefined ultrasound composite and considered a thyroid nodule as positive when two or more of the five suspicious features, irregular margin (M), microcalcification (C), hypoechogenicity (H), increased vascularity (V), and suspicious cervical lymph node (L), were present. This rule demonstrated high sensitivity but only moderate specificity for detecting malignant thyroid nodules in this retrospective study. While the combination of irregular margins and microcalcifications was strongly associated with malignancy, these ultrasound findings should be interpreted alongside FNAC results and clinical judgment rather than used independently to guide surgical decisions. Before any broader clinical application, the diagnostic rule requires validation in prospective, non-surgical populations, assessment of inter-observer agreement, AUC reporting with confidence intervals, and comparison with established TI-RADS and ATA systems.
